# A Scientometric Visualization Analysis for Molecular Mechanisms of Substance Abuse and Its Neurotoxicity From 1997 to 2021

**DOI:** 10.3389/fnmol.2022.885701

**Published:** 2022-07-01

**Authors:** Aijia Zhang, Zilong Liu, Man Liang

**Affiliations:** Department of Forensic Medicine, Tongji Medical College, Huazhong University of Science and Technology, Wuhan, China

**Keywords:** scientometric analysis, visualization, molecular mechanism, substance abuse, neurotoxicity

## Abstract

Substance abuse has become a global problem due to drug-induced addiction and neurotoxicity, which causes a huge physical, social, and financial burden. Various kinds of drugs can hijack the users’/abusers’ behavior and associated neurocircuitry. To summarize recent scientific advances on drug abuse, we reviewed relevant publications to analyze research progress and such trends through bibliometric ways. Based on retrieval strategies, a total of 681 scientific records published from 1997 to 2021 were screened and included in the Web of Science (WoS) database. Further scientometric analysis revealed that annual publication output increased across this period, with the United States of America (USA) contributing a significant number of reasons. Research has focused on neurotransmitter, oxidative stress, mitochondrial system injury, and other neurotoxic mechanisms. Neuroimmune, neurotoxic targets, and new psychoactive substances have been hot topics in recent years, which deserve continued research in the future. Specific research on molecular mechanisms has progressed across this period, with an emphasis on the root cause of toxicity and molecular targets for therapy. Moreover, collaborations of international multi-disciplinary research teams have been efficient and need to be encouraged for addiction research and the development of appropriate therapeutic processes.

## Introduction

Substance abuse, including relapse, refers to excessive consumption of stimulants, cannabis, amphetamines, cocaine, and hallucinogens, according to ICD-11 (WHO, 2021) and DSM-5 (Quinn et al., 1997; Büttner and Weis, 2004; Cassel and Bernstein, 2008; American Psychiatric Association [APA], 2013), which induces physical and/or psychological harm, deteriorating cognition and motivation, as well as impulsive and aggressive behavior (Majewska, 1996; Pau et al., 2002). Addicts suffer from complex neurological impairments that are often associated with neurodegenerative diseases, such as Parkinson’s disease (Cadet and Krasnova, 2009; Graves et al., 2021). According to the report of the European Monitoring Centre for Drugs and Drug Addiction (EMCDDA) in 2020, both the number of drug-related deaths and the number of addicts, across the European Union (EU), are increasing, which aggravates the global burden on public health systems, resulting in huge worldwide economic costs, including law enforcement resources (Rehm et al., 2009; EMCDDA, 2020).

Studies have shown that abused drugs activate the mesolimbic dopaminergic system, orbitofrontal cortex, and extended amygdala (Cunha-Oliveira et al., 2008; Koob and Volkow, 2010; Chen et al., 2021a,b). Specifically, substance abuse alters intracellular signaling pathways, transcription factors, and levels of gene expression in these reward circuits (Nestler, 2004; Hyman et al., 2006; Cunha-Oliveira et al., 2008; Koob and Volkow, 2010; Liao et al., 2021). In addition, recent advances have highlighted psychoactive substance-induced neuroinflammation, blood–brain barrier (BBB) dysfunction, and neurogenesis deficits (Gonçalves et al., 2014; Crews et al., 2021).

To provide an international description of scientific activities examining substance abuse, a bibliometric analysis is needed to quantify the influences of scientific literature sets, academic characteristics (e.g., journals), institutions, highly cited literature, high-frequency identifiers, and the relevant clustering of the above. The visible technology that provides an overview of programmatic research and its developmental trends will provide researchers with a clearer understanding of relative research breakthroughs and their expected future dynamics (Kamdem et al., 2019). With this in mind, we review the research on the neurotoxicity of substance abuse and relapse with the goal of deepening our understanding of this progress as well as trends for future research, and molecular targets for therapeutic development.

## Materials and Methods

**FIGURE 1:**
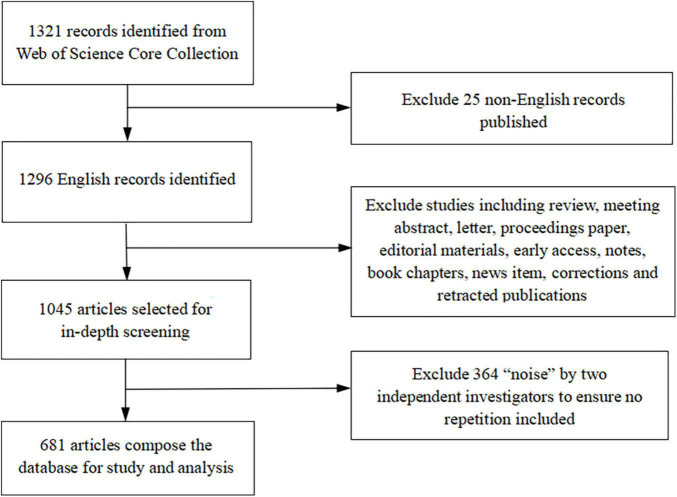
The flowchart of methodology.

The Medical Subject Headings (MeSH) listed in PubMed were applied to obtain synonyms and the following search strategy: [Topic#3 = (Topic#1) OR (Topic#2)], {Topic#1 = [(“Substance Abuse”) OR (“Substance Dependence”) OR (“Substance Addiction”) OR (“Substance Habituation”) OR (“Drug Abuse”) OR (“Drug Dependence”) OR (“Drug Addiction”) OR (“Drug Habituation”)] AND [(“Neurotoxicity”) OR (“Neurotoxic Effect”)]}, {Topic#2 = [(“Relapse of Substance Abuse”) OR (“Relapse of Substance Dependence”) OR (“Relapse of Substance Addiction”) OR (“Relapse of Substance Habituation”) OR (“Relapse of Drug Abuse”) OR (“Relapse of Drug Dependence”) OR (“Relapse of Drug Addiction”) OR (“Relapse of Drug Habituation”)] AND [(“Neurotoxicity”) OR (“Neurotoxic Effect”)]}. The Web of Science (WoS) Core Collection database is a global academic database indexing more than 20,000 authoritative and high-impact academic journals, covering natural science, engineering technology, biomedicine, social science, and arts and humanities. We retrieved 1,321 records published between January 1997 and December 2021, and excluded 25 non-English records, narrowing the list to 1,296 English manuscripts for further analysis. A total of 1,045 original articles were identified, and studies, including review, meeting abstract, letter, proceedings paper, editorial materials, early access, notes, book chapters, news items, and corrections, were excluded. Two investigators screened all of the original data, eliminated noise, respectively, and reached a consensus *via* discussion and re-evaluation by a third investigator when discrepancies were observed. Finally, 681 articles were assembled for scientometric mining (Figure 1). We used CiteSpace 5.8.R3 for statistical analysis and developed visual charts, including publication output flows, cooperation agreements between countries, co-occurrent observations, and keyword/identifier bursts for mapping research trends and frontiers (Chen, 2004, 2006; Chen et al., 2010, 2014).

## Results

### Publication Trends

**FIGURE 2:**
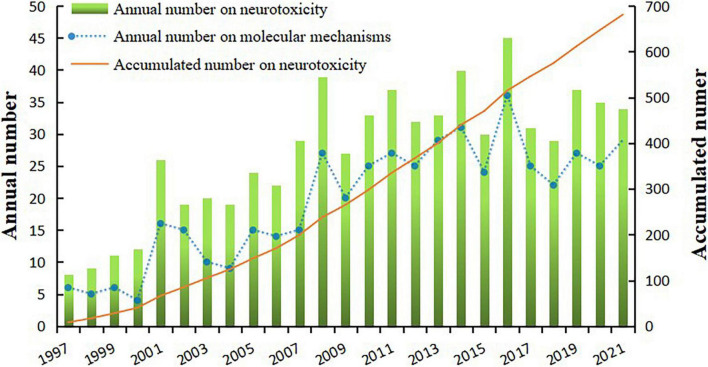
Annual number and accumulated number of articles on neurotoxicity of substance abuse, and number of publications on molecular mechanisms from 1997 to 2021.

Overall, annual publication outputs had a generally positive growth rate, except for a few fluctuations, since 1997; for this period, the peak annual publication count was 45 in 2016 (Figure 2). In line with an increasing number of annual publications, substance abuse and its neurotoxicity remained an important research topic across this time period. Using the Mann–Kendall (MK) test, the statistical results (*Z* = 4.2099 > 2.32 > 0) indicated a significant increasing trend of annual publication outputs related to neurotoxicity of substance abuse with a 0.001 level of significance. Remarkably, according to the categorization of the topics, 487 publications were focused on molecular mechanisms, while the rest were almost all on behavior or neuropsychology. Accordingly, the proportion of articles on molecular mechanisms also increased year by year, with its highest level in 2013 at 87.88% (29/33, Figure 2).

### Contributing Countries and Institutions

**FIGURE 3:**
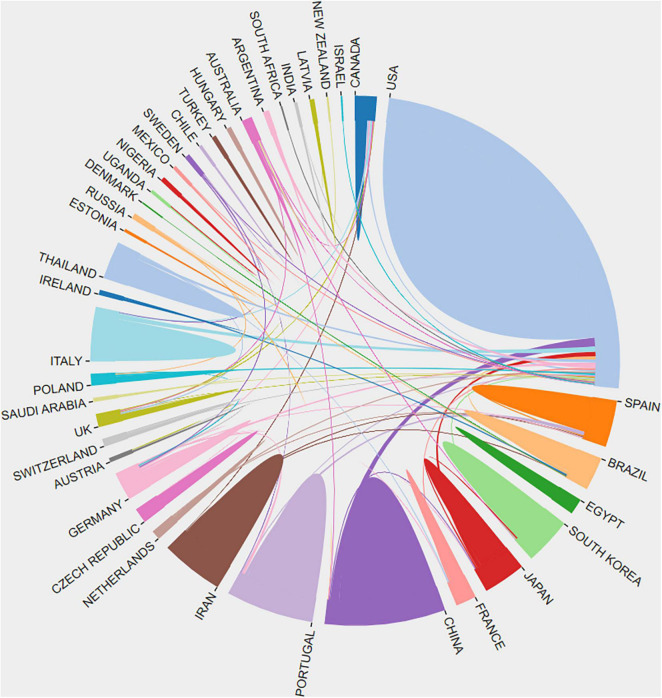
Cooperation between contributed countries.

Overall, 44 countries/regions contributed scientific outputs with intensive bilateral cooperation during this period. The United States (50.81%, 346/681) was the most productive country, exhibiting a marked excess in volume of research output, followed closely by the People’s Republic of China (9.25%, 63/681), Spain (7.93%, 54/681), and Japan (4.85%, 33/681) (Table 1 and Figure 3). Besides this, other Asian (20.26%, 138/681) and European (23.49%, 160/681) countries have also published more in recent years. Among these top 10 productive countries, United States had the greatest number of publications devoted to studies on molecular mechanisms of neurotoxicity and substance abuse (67.05%, 232/346), followed by Portugal (83.33%, 25/30).

**TABLE 1:** The top 10 productive countries and institutions.

Country or region	Articles (%)	Citations	H-index	Citations per article	Top country institution	Top institution articles (%)
United States	346 (50.81%)	15023	61	43.42	National Institutes of Health Nih United States	52 (15.03%)
Peoples R China	63 (9.25%)	861	18	13.67	Southern Medical University China	9 (14.29%)
Spain	54 (7.93%)	1436	24	27.09	University of Barcelona	16 (29.63%)
Japan	33 (4.85%)	1062	16	32.18	Chiba University	5 (15.15%)
Germany	30 (4.41%)	1355	20	45.17	Humboldt University of Berlin	9 (30.00%)
Portugal	30 (4.41%)	984	18	32.80	Universidade Do Porto	27 (90.00%)
Italy	28 (4.11%)	759	14	27.11	University of Cagliari	7 (25.00%)
Iran	24 (3.52%)	173	8	7.21	Shahid Beheshti University Medical Sciences	8 (33.33%)
England	18 (2.64%)	698	13	38.78	University of London	4 (22.22%)
South Korea	18 (2.64%)	353	8	19.61	Kangwon National University	7 (38.89%)

Figure 3 also depicts bilateral cooperation between contributing countries; the United States cooperated with 22 countries, making it the most collaborative country, followed by Germany and Canada, each, respectively, cooperating with 6 other countries. This indicates that the countries/regions shared and facilitated greater increase in the research field of substance abuse by exponential collaborations.

### Highly Cited Publications

The 681 articles received a total of 22,625 citations according to the WoS, giving an average citation of 33.22 per article (22,625/681). Without a defined conservative cutoff of time limit for citation, Germany (45.17, 1355/30), the United States (43.42, 15023/346), and England (38.78, 698/18) were the top 3 leaders in citations per article. Other than this, the average H-index of the retrieved papers queried on the WoS was 71; and the top 3 countries were the United States with the highest H-index of 61, followed by Spain (24) and Germany (20). Furthermore, the core institutions in the countries, such as Portugal (90.00%), South Korea (38.89%), and Iran (33.33%), had other national achievements (Table 1).

Table 2 lists the top 10 most-cited articles, which were regarded as high-quality scientific research published in authoritative journals categorized in psychiatry (No. 1, 9), neuroscience (No. 2, 3, 7, 10), nature medicine (No. 4), neurology (No. 5, 6), and preventive medicine (No. 8). These most cited papers examined the mechanisms of psychomotor impairment and neurotoxicity, and eight of the top 10 papers examined neurotoxic molecular mechanisms, like a role of the dopamine transporter (Volkow et al., 2001; Munro et al., 2006). Impressively, nine of the top 10 articles were contributed by the United States, and another one was contributed by England; correspondingly, IF5 and SJR of these top two countries were the United States (IF5: 17.825, SJR: 5.477) and England (IF5: 49.248, SJR: 19.536), respectively.

**TABLE 2:** The characteristics of highly cited articles.

Rank	Total citations	Article title	Journal	Published year	Country	IF 2020	IF 5- years	SJR 2020
1	688	Association of dopamine transporter reduction with psychomotor impairment in methamphetamine abusers	American Journal of Psychiatry	2001	United States	18.112	17.825	5.477
2	459	Reduced striatal dopamine transporter density in abstinent methamphetamine and methcathinone users: Evidence from positron emission tomography studies with [C-11]WIN-35,428	Journal of Neuroscience	1998	United States	6.167	6.993	3.483
3	437	Loss of dopamine transporters in methamphetamine abusers recovers with protracted abstinence	Journal of Neuroscience	2001	United States	6.167	6.993	3.483
4	423	Nitrous oxide (laughing gas) is an NMDA antagonist, neuroprotectant and neurotoxin	Nature Medicine	1998	England	53.440	49.248	19.536
5	277	Memory impairment in abstinent MDMA (”Ecstasy”) users	Neurology	1998	United States	9.910	10.664	2.910
6	275	Evidence for long-term neurotoxicity associated with methamphetamine abuse–A H-1 MRS study	Neurology	2000	United States	9.910	10.664	2.910
7	275	Small changes in ambient temperature cause large changes in 3,4-methylenedioxymethamphetamine (MDMA)-induced serotonin neurotoxicity and core body temperature in the rat	Journal of Neuroscience	1998	United States	6.167	6.993	3.483
8	249	Sex differences in striatal dopamine release in healthy adults	Biological Psychiatry	2006	United States	13.382	14.103	5.335
9	245	Methamphetamine causes microglial activation in the brains of human abusers	Journal of Neuroscience	2008	United States	6.167	6.993	3.483
10	210	Neuronal apoptosis associated with morphine tolerance: Evidence for an opioid-induced neurotoxic mechanism	Journal of Neuroscience	2002	United States	6.167	6.993	3.483

### Active Journals

More than 200 authoritative journals published articles on the neurotoxicity of substance abuse. Among these, the top 10 of these active journals contained 28.93% of the manuscripts, such as Neurotoxicity Research (3.82%, 26/681), Neuroscience (3.67%, 25/681), and Psychopharmacology (3.52%, 24/681) (refer to Table 3); fields of research included neuroscience, biochemistry, pharmacology, as well as toxicology and pharmaceutics.

**TABLE 3:** The 10 most active journals that published articles on substance abuse and its neurotoxicity research.

Journal	Published numbers (%)	IF 2020	SJR 2020	JCR quartile	Categories
Neurotoxicity Research	26 (3.82%)	3.911	0.923	Q2	Neuroscience; pharmacology, toxicology and pharmaceutics
Neuroscience	25 (3.67%)	3.590	1.297	Q3	Neuroscience
Psychopharmacology	24 (3.52%)	4.530	1.378	Q2	Pharmacology; toxicology and pharmaceutics
Brain Research	20 (2.94%)	3.252	1.037	Q3	Biochemistry, genetics and molecular biology; medicine; neuroscience
Neuroscience Letters	19 (2.79%)	3.046	0.944	Q3	Neuroscience
Neuropharmacology	18 (2.64%)	5.251	1.760	Q2	Neuroscience; pharmacology, toxicology and pharmaceutics
Neuropsychopharmacology	18 (2.64%)	7.855	2.704	Q1	Medicine; pharmacology, toxicology and pharmaceutics
Neurotoxicology	16 (2.35%)	4.294	1.060	Q2	Neuroscience; pharmacology, toxicology and pharmaceutics
Journal of Neurochemistry	16 (2.35%)	5.372	1.750	Q2	Biochemistry, genetics and molecular biology; neuroscience
Pharmacology Biochemistry and Behavior	15 (2.20%)	3.533	1.184	Q2	Biochemistry, genetics and molecular biology; neuroscience; pharmacology, toxicology and pharmaceutics

### Hotspots of Research

**FIGURE 4:**
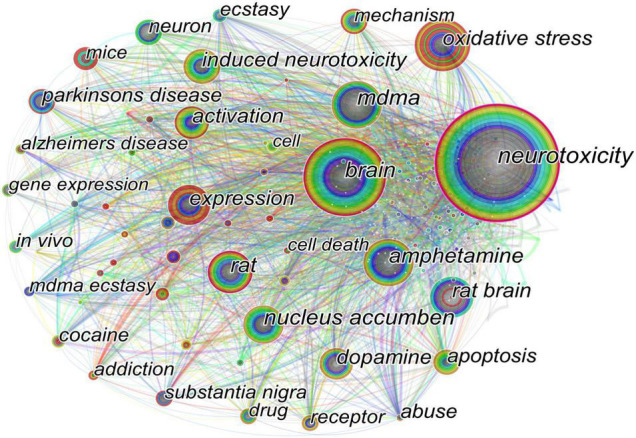
Network of keywords co-occurrence. The circle size and the link illustrated the frequency and relevance of keywords, respectively.

After excluding irrelevant and repeated keywords, we analyzed, merged, and visualized the network maps of keyword co-occurrence by CiteSpace (Figure 4), which illustrated the relevance and frequency of keywords using circle link and size. High-frequency keywords, including “neurotoxicity” (frequency: 188, centrality: 0.23), “brain” (frequency: 117, centrality: 0.13), “oxidative stress” (frequency: 79, centrality: 0.09), “rat” (frequency: 77, centrality: 0.11), and “mdma” (frequency: 75, centrality: 0.11), represented by larger circles. Other keywords, such as “amphetamine” (frequency: 71, centrality: 0.10), “dopamine” (frequency: 62, centrality: 0.09), “nucleus accumbens (NAc)” (frequency: 57, centrality: 0.13), “activation” (frequency: 53, centrality: 0.08), and “apoptosis” (frequency: 42, centrality: 0.05) showed moderate frequencies.

### Scientific Landscapes and Trends

**FIGURE 5:**
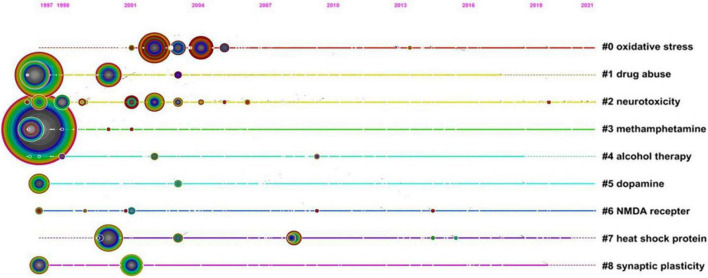
Co-cited references timeline map of neurotoxicity of substance abuse. Nodes represent referenced documents. The size of nodes represents the frequencies of cited references, and the location reflects the present time. Years are arranged horizontally at the top. The clusters are performed based on the themes of co-cited references and the label of each cluster is shown at the end of the timeline.

After clustering and mapping co-cited literatures (Figure 5), we analyzed the timeline of developing trends of specific research fields, the observed shift of research focus, and the formal professional structures of these research systems (Niazi and Hussain, 2011). According to an in-depth analysis of the top ten clusters, we identified that most were distributed in the period from 1997 to 2010. The earlier studies have focused on methamphetamine (#3), drug abuse (#1), and dopamine (#5); then, the research hotspots shifted to heat shock protein (#7), synaptic plasticity (#8), NMDA receptor (#6), oxidative stress (#0), and neurotoxicity (#2), which are all involved in neurotoxic molecular mechanisms of substance abuse. As for alcohol therapy (#4), a therapeutic implication of drug abuse has maintained researchers’ attention for several years.

**FIGURE 6:**
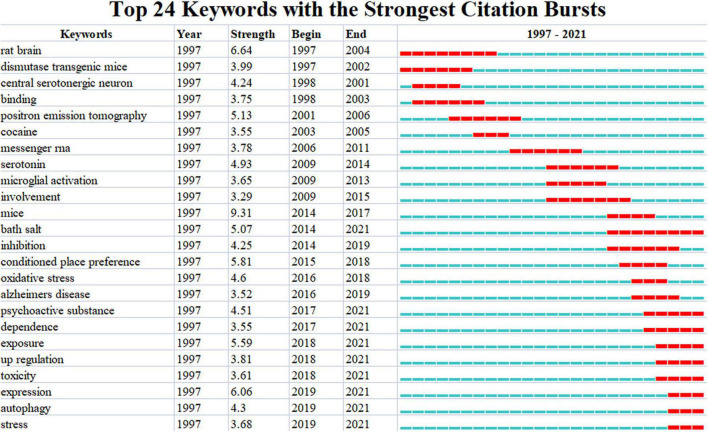
Top 24 keywords with the strongest citation bursts. Each blue or red short line represents a year, and a red line stands for a burst detected year.

We found that keywords could reveal research topics and contents, which play an apparent and updated role in tracking the transition of research hotspots. We obtained a keyword burst map to visualize shifts in focus and emergence across time on the basis of the strongest burst of keywords in scientific research articles (Figure 6). In addition, burst keywords could summarize outstanding research topics over any period of time by indicating the duration and intensity of these hot issues (Kleinberg, 2003; Chen, 2004, 2006; Chen et al., 2010, 2014). According to the top 24 keywords with the strongest citation bursts, the earlier research hotspots were neurotoxic target-organs and animal models (dismutase transgenic mice, rat brain, and central serotonergic neuron), examination (positron emission tomography), and commonly abused drugs (cocaine). Then, the burst keywords shifted to molecular mechanism-related items (e.g., messenger RNA, serotonin, microglial activation, oxidative stress, autophagy) and new psychoactive substances (e.g., bath salt and psychoactive substance). Besides these, the relationship between substance abuse and Alzheimer’s disease and some comorbid models were examined.

## Discussion

Our study performed a visualized bibliometric analysis of substance abuse and its neurotoxicity from 1997 to 2021 using CiteSpace. Substance abuse has caused increasing global harm by impairing the nervous system and the development of psychiatric symptoms (Şenel and Demir, 2018; EMCDDA, 2020; da Silva et al., 2021). More and more research has focused on the neurotoxic progression of substance abuse, especially the complexities of molecular mechanisms. These fields of research have needed a review to map the publications providing new ideas for the treatment and prevention of drug abuse and relapse (i.e., hot topics).

Drug abuse has brought a tremendous socioeconomic burden to the whole world, especially in recent decades (Hagemeier, 2018); this dreadful situation needs more research to expose the severity of drug addiction and drug-induced neurological disorders. Encouragingly, from 1997 to 2021, the total amount of related publications reached a total of 681, with an average of 27 per year; and an annual number of publications had a generally rising trend with a peak in 2016.

Over the past 25 years, the United States, contributing more than half of the total publications, has played a leading significant role in productivity in this field, and Asian and European countries came followed with far less production. Regarding the reason for this status, in the United States, various types of smuggled drugs have flooded its streets; meanwhile, classic opiates, cocaine, and amphetamines were pursued by a large number of substance abusers. Except for such a severe social landscape, the United States government offered abundant research funds to support high-level research activity, which resulted in a high academic reputation and characteristic H-index value in scientific research (e.g., Substance Abuse and Mental Health Services Administration, 2002). Among the European countries, Germany published a medium amount of research and had the highest number of citations per article, which indicated significant researchers’ close attention.

Considering its global harm, there has been a significant increased hot topic research and collaborative projects in countries, such as the United States, Germany, and Canada (Fujáková-Lipski et al., 2017; Costa et al., 2021; Sogos et al., 2021). Based on legalized marijuana policies in the United States and Canada, similar domestic and international situations and potential cooperative opportunities increased exponentially; additionally, the United States provides attractive financial grants to facilitate efficient cooperation and productivity (Goundar et al., 2021; Isfandyari-Moghaddam et al., 2021). Under a shared global concern of drug abuse, worldwide scientific cooperation should be further strengthened to highlight research quality, and the outcomes should be noted to more deeply illustrate the harm of substance abuse and drug-induced toxicity (Rahim et al., 2012).

The 10 most active journals, as shown in Table 3, were of high quality and rational JCR quartile on neuroscience and neuropsychiatry, published nearly one-third of the total publications. Of the molecular mechanisms of neurotoxicity of substance abuse, the understanding has reached unprecedented depth, and significant advances are laudable over the past few decades. The neurotoxic effects of drug abuse are associated with oxidative stress, mitochondrial dysfunction, apoptosis, and neurogenesis inhibition, directly or indirectly affecting neurotransmitter systems, including but not limited to dopaminergic and glutamatergic neurons, and causing irreversible neuronal damage (Cunha-Oliveira et al., 2008; da Silva et al., 2021).

The bibliometric indicators, including publication numbers, citations per article, journal impact factors, crown indicators, H-index, and its variants, can manifest high-quality scientific results (Joshi, 2014). Our results indicated that the average citation per article on the neurotoxicity of substance abuse was 33.22, higher than on other subjects, implying that the topic has kept a strong research interest. In general, highly cited articles are published in journals with high SJR or IF (Falagas and Alexiou, 2008). Notably, four of the top 10 most-cited articles have garnered more than 400 citations, and nine articles were published in journals with IF >5 or higher. From the angle of contributing countries, nine literatures were ascribed to the United States, which constituted a major force in this field; and the other one, published in Nature Medicine, the only journal with IF >50, was authored by researchers from England.

Furthermore, six of these articles focused on studies related to methamphetamine, while the remaining four articles analyzed the neurotoxicity mechanisms of 3,4-methylenedioxymethamphetamine (MDMA), nitrous oxide (laughing gas), and neuronal apoptosis associated with morphine tolerance. This could be interpreted that methamphetamine, as one of the most widely abused drugs, has attracted more researchers’ interests than others; and the mainstream views show that significant alterations in dopamine and serotonin by dopamine transporter dysfunction in the striatum led to long-term neuronal damage and ultimately motor and cognitive impairment (Volkow et al., 2001). Among the methamphetamine abusers, the aforementioned neurotoxicity is significantly more severe in men than in women; nevertheless, no significant molecular differences were observed between the sexes (Munro et al., 2006). Moreover, the 10 most-cited articles were all published from 1998 through 2008, which suggest that these results have primarily progressed and verified the mechanisms of neurotoxicity of substance abuse in this decade and enlightened more research afterward.

According to the presented keyword co-occurrence network, the core direction of the specific research topics was examined. In Figure 4, the clustered topics, such as neurotoxicity, brain, oxidative stress, dopamine, MDMA (ecstasy), amphetamine, Parkinson’s disease, and Alzheimer’s disease, can be categorized as “neuroscience,” “molecular biology,” “epidemiology,” or “psychoneuropathology”; and these interdisciplinary aspects represent the evolutionary process of more concerning issues in this field.

The neurotoxic effects of abused substances could be described as inhibiting (e.g., opioids, cannabinoids, nicotine) or stimulating (e.g., cocaine and amphetamines) neurotransmitters, decreasing dopamine and serotonin reserves by inactivating their transporters, reducing vesicular monoamine transporters, and activating alpha-(2)-adrenergic receptors (Volkow et al., 2001; Cozzi and Foley, 2003; Garrido et al., 2005; Jîtcã et al., 2021). Besides, studies also proved that tachykinins, especially substance P (SP), are linked to the terminal degeneration of dopamine through NK-1 receptor activity in substantia nigra for chronic methamphetamine abusers (Yu et al., 2002). The neurotoxicity and senescence induced by methamphetamine resulted from the upregulation and aggregation of presynaptic protein α-synuclein (Fornai et al., 2005; Wu et al., 2021). Of note, drug participatory induced the release of monoamines from neuronal storage vesicles, metabolism by monoamine oxidase or catechol-O-methyltransferase (COMT), and reactive oxygen species (ROS) generation (da Silva et al., 2021); meanwhile, hydrogen peroxide, as a by-product of these activities interacting with transition metal ions, generates toxic hydroxyl radicals, which indicate that drug-induced excessive neurotransmitter release has a strong correlation with oxidative stress (Barbosa et al., 2012; Graves et al., 2021). It is worth noting that drug-induced mitochondrial metabolism dysfunction promotes oxidative stress by inhibiting oxidative phosphorylation and ATP production, triggering apoptotic signaling pathways, and then causing neuronal damage (Burrows et al., 2000; Huang et al., 2019). It has been shown that methamphetamine and cocaine, interfering with all electron transport chain (ETC) complexes, producing excess ROS, further aggravate mitochondrial disintegration, while mephedrone affects the activity of mitochondrial complex II and IV (Yang et al., 2018; Naserzadeh et al., 2019; Thornton et al., 2021).

Besides the above, the highly cited articles demonstrated that abused drugs could increase microglial reactivity and lead to neuroinflammation, which has shown a strong link to neurotoxicity (Lacagnina et al., 2017). Amphetamine compounds, acting on dopamine D1-like receptors, microglia, and relevant signaling proteins, exacerbated neuroinflammation responses and promoted macrophage polarization from M0 to M1, which provides a potential basis of long-term neurotoxicity (Li et al., 2018; Wang et al., 2019). Cocaine activated microglia in NAc, increasing the production and release of tumor necrosis factor-alpha (TNF-α) (Lewitus et al., 2016). Cannabis could impair the activation of Toll-like receptors 4 (TLR4) by inhibiting cytokine production and interfering with TLR-induced immune responses (Sexton, 2020). Moreover, synthetic cathinone and ketamine have been confirmed to induce the release of pro-inflammatory cytokines and microglial activation (Li et al., 2017; Oliver et al., 2018; Erickson et al., 2019). Regarding methamphetamine abuse, melatonin could reduce the expression of pro-inflammatory cytokines, inhibit the expression of inflammation-mediated enzymes, such as TNF-α and iNOS, and suppress neuroinflammation (Deng et al., 2006; Olcese et al., 2009; Veneroso et al., 2009). These referenced molecular mechanisms may provide valid and reliable therapy potential for substance abuse and relapse in the future.

As a prominent feature of bibliometrics, the research topic clustering technology categorizes all retrieved records with similar topics and generates a timeline map (Waltman et al., 2010). According to the clustering of the co-cited literatures, NMDA receptor (#6, *N*-methyl-D-aspartic acid receptor) has been a significant focus for a long time. Based on glutamatergic neurotransmission involved in several substances of abuse, activated NMDA receptors would increase intracellular calcium concentration, which increases extracellular glutamate with positive feedback (Rego and Oliveira, 2003; Tzschentke and Schmidt, 2003). Methamphetamine increased glutamate by exocytosis of astrocytes through TNF production and calcium mobilization, which thereby promoted microglial reactivity to neuroinflammation, and neuronal death (Canedo et al., 2021). In addition, other psychoactive substances (e.g., morphine, cocaine, and heroin) could increase extracellular glutamate concentrations in the ventral tegmental area (VTA), NAc, prefrontal cortex, and striatum (Siggins et al., 2003; Williams and Steketee, 2004; Domingues et al., 2006; You et al., 2007). Otherwise, NMDA receptor antagonists, memantine and MK-801, may inhibit physical dependence and tolerance (Tzschentke and Schmidt, 1995; Trujillo, 2000; Ribeiro Do Couto et al., 2004). Therefore, further research on NMDA receptor activities may provide new strategies to treat substance abuse.

By CiteSpace algorithm-dependent keyword citation burst detection (Chen, 2006), compared with traditional drugs, new designer substances, such as “bath salt” from 2014, containing one or more synthetic cathinone derivatives, such as the most popular components of 4-methylcathinone (mephedrone), 3,4-methylenedioxymethcathinone (methylone), and 3,4-methylenedioxypyrovalerone (MDPV) (Baumann et al., 2013), have become popular particularly among adolescents since the mid-2000s (Altun and Çok, 2020). Similar to traditional drugs, synthetic cathinones also act on monoaminergic terminal functional markers, increasing ROS, activating apoptotic signaling pathways, and causing cytotoxicity (Leyrer-Jackson et al., 2019).

According to the bibliometrics analysis, we found that research on poly drug abuse often co-occurred recently. As quite a few drug abusers may prefer multi-substances of abuse (Gerostamoulos et al., 2001; Preti et al., 2002; Coffin et al., 2003; Darke, 2003; Steentoft et al., 2006), more studies focused on synergistic effects of alcohol and psychoactive substances; e.g., recreational co-abuse of alcohol and ketamine could aggravate characteristic apoptosis in morphologically sub-G1 phase and lead to intra-neuronal Ca^2+^ overload, down-regulation of p-Akt, p-CREB, PKA, CaMK-IV, Bcl-2, and BDNF, and up-regulation of cleaved caspase-3 and Bax (Moore et al., 1997; Wu et al., 2006; Quek et al., 2013; Zuo et al., 2017).

Other proportionately interesting findings on the neurotoxicity of substance abuse were also retrieved. Curcumin, a natural polyphenol extracted from the rhizome of *Curcuma longa* L, and *Melissa officinalis*, an antioxidant plant, both scavenge oxygen free radicals and inhibit monoamine oxidase to antagonize drug-induced neuronal apoptosis (Hassanzadeh et al., 2011; Ryskalin et al., 2021). This suggests a potential neuroprotective role of natural plant extracts for the treatment of substance abuse. A number of pharmacological interventions for drug-related memory deficits have shown significant benefits in preventing relapse at the preclinical level, such as propranolol interfering with heroin memory consolidation and reducing subsequent drug-seeking, making it an attractive treatment candidate for opioid addiction and relapse prevention (Chen et al., 2021a,b). Moreover, mTOR (mammalian target of rapamycin) interferes with cocaine-related memory consolidation, reduces cocaine-seeking behavior, and prevents relapse, and these effects are extract-dependent and time-specific (Zhang et al., 2021). Besides, 5-hydroxytryptamine receptor 1F (5-HT1F) receptor agonist, LY 344864, and cannabidiol showed a therapeutic prevention of relapse to drug addiction (Shahidi et al., 2018; Metz et al., 2021).

## Conclusion

While there has been an exponential increase in substance abuse harm and corresponding research, there is still a paucity of studies examining systematic bibliometric progress. With the included visualized illustration, we constructed this assessment to elucidate interdisciplinary information of research on psychoactive substances. Based on the studies of neurotoxicity, pharmacological effects, and epidemiology, this update on research evidence suggests greater scientific therapeutic predictability to combat addiction to psychoactive substances. The overall findings of this study suggest that research hotspots have focused on molecular mechanisms, and more scientific priority should be given to allocate research resources for these studies with the greatest potential to investigate therapeutic applications.

## Limitations

There were inevitable limitations to our study. First, since the scientific literature database (WoS) keeps dynamic publishing, the interval lag between the publication and the retrieval would affect the time sensitivity of studies. Second, to comply with the software condition of CiteSpace, only English studies were analyzed for the function of co-occurrence and co-citation analysis methods (incompatibility of multiple languages).

## Data Availability Statement

The original contributions presented in this study are included in the article/supplementary material, further inquiries can be directed to the corresponding author.

## Author Contributions

ML and ZL contributed to the conception, design of the study, reviewed, and edited the manuscript. AZ organized the database and performed the statistical analysis. AZ and ML wrote the first draft of the manuscript. All authors contributed to the manuscript revision, read, and approved the submitted version.

## Conflict of Interest

The authors declare that the research was conducted in the absence of any commercial or financial relationships that could be construed as a potential conflict of interest.

## Publisher’s Note

All claims expressed in this article are solely those of the authors and do not necessarily represent those of their affiliated organizations, or those of the publisher, the editors and the reviewers. Any product that may be evaluated in this article, or claim that may be made by its manufacturer, is not guaranteed or endorsed by the publisher.
